# Cold Plasma-Treated Chickpea Protein Isolate: Effects on Rheological Behavior and Quality Characteristics of Allergen-Free Rice Muffins

**DOI:** 10.3390/foods14213635

**Published:** 2025-10-24

**Authors:** Jiayu Sun, Jian Wang, Zimo Wen, Ye Liu, Daodong Pan, Lihui Du

**Affiliations:** State Key Laboratory for Managing Biotic and Chemical Threats to the Quality and Safety of Agro-Products, Zhejiang-Malaysia Joint Research Laboratory for Agricultural Product Processing and Nutrition, College of Food Science and Engineering, Ningbo University, Ningbo 315832, China

**Keywords:** allergen-free muffins, chickpea protein isolate, cold plasma, rheological properties, quality properties

## Abstract

Allergen-free (AF) baked goods usually show inferior texture and mouth-feel due to lack of functional proteins. This study evaluated the quality characteristics of AF muffins incorporated with three different sources of chickpea protein isolate (CPI), including commercial CPI, laboratory CPI, and cold plasma-modified laboratory CPI at varying addition levels (5%, 10%, and 15%). Results indicate that commercially available CPI exhibits high viscoelasticity in whole wheat muffin batter due to mixed protein types and severe denaturation, but the finished muffins are excessively hard with insufficient elasticity. Adding 15% laboratory CPI treated with cold plasma significantly enhanced the viscoelasticity of the muffin batter. The final product achieved a volume of 99.43 cm^3^, representing a 20.1% increase compared to the protein-free control group. This resulted in a superior product with enhanced elasticity, moderate hardness, and improved color. This study confirms that cold plasma modification technology effectively unlocks the structural and functional potential of chickpea protein in AF baking systems, offering an innovative solution for developing high-quality, high-protein AF foods. Future research will focus on the industrial scalability of this technology, product sensory characteristics, and shelf-life evaluation.

## 1. Introduction

According to the FAO [1], approximately 220 million people worldwide suffer from food allergies (FA). They affect about 8% of children and 10% of adults globally, with prevalence continuing to rise each year [2,3]. Severe FA can lead to anaphylactic shock, which poses a serious threat to human life and health. Currently, there is no cure for FA; the only approach is to avoid allergens or provide acute medical treatment. Presently, more than 160 foods are recognized to trigger FA [4,5]. Among these, cereals containing gluten, eggs, milk, peanuts, fish, crustacean shellfish, sesame, and tree nuts are recognized by the WHO and FAO as the eight common allergens [6,7]. Patients with FA should strictly avoid consuming certain foods and substitutions in their daily foods are necessary. Therefore, the demand for low-allergen foods is growing, creating a large market, such as for gluten-free [8,9] and egg-free foods [10]. In recent years, the market for foods that do not contain the eight major allergens, also known as allergen-free (AF) foods, has been expanding, as it has been reported that more than one-third of people with FA are allergic to more than one type of allergen [6].

AF baked goods, such as bread, cakes, and cookies, pose a significant challenge in product development due to the inability to use wheat, eggs and milk, which provide key functional proteins essential for the structure and texture of the baked products [11]. After removing traditional ingredients such as wheat flour and eggs, AF baked goods are often challenged by textural defects such as high hardness and uneven porosity, and reduced processing performance such as insufficient batter viscoelasticity and poor bubble stability [12]. However, the development of AF foods currently faces a core contradiction: on one hand, non-allergenic plant proteins are frequently needed to construct gluten-like network structures; on the other hand, their functional properties are often inadequate to replace traditional wheat or milk proteins [13,14]. Currently, the primary ingredients in AF baked goods are still plant-based components, including various starches, modified starches, proteins, and fibers [15]. This over-reliance on starchy ingredients, while partially supporting the product structure, leads to two significant issues: firstly, the final product’s texture and mouthfeel are often unsatisfactory; secondly, it reduces the nutritional value of the product, potentially leading to inadequate protein intake for individuals with allergies [16]. Therefore, improving the functional properties of plant-based proteins used in AF foods is essential for enhancing the quality of these baked products while also increasing their protein content. Despite its significance, research on this topic is still very scarce.

Physical and chemical modifications are typical approaches for functional improvement of plant proteins. While traditional modification techniques like enzymatic hydrolysis, extrusion, or ultrasonication are effective, they present certain limitations such as high cost, potential nutrient loss during processing, or limited modification efficacy [17]. In contrast, cold plasma (CP) treatment, as an emerging non-thermal physical modification technology, offers unique advantages such as high processing efficiency, low energy consumption, no requirement for chemical reagents, and no residual byproducts. CP contains abundant high-energy reactive oxygen and nitrogen species. These active substances can interact with proteins, unfold the structure of proteins, expose buried hydrophobic residues, modify amino acid side chains, and promote aggregation and cross-linking under certain CP parameters, making protein structures more orderly, therefore enhancing the ability of proteins to form gels and stabilize emulsions [18]. Our previous research has demonstrated that CP treatment can effectively enhance the functional properties of plant proteins, such as pea protein isolate and chickpea protein isolate (CPI), improving characteristics like foaming ability, emulsifying properties, and gelling properties [18,19]. CPI offers distinct advantages as an AF ingredient, including low allergenicity, high bioavailability, reduced bitterness, and a rich lysine content, yet it has not been widely studied in AF baking formulations [20]. Shaabani et al. developed a gluten-free muffin formulation using unmodified CPI, along with transglutaminase and whole egg, to form the desired gel network [21]. However, that study did not address the challenge of unmodified CPI forming a gel network on its own in a strictly AF system devoid of eggs and milk [22]. Therefore, investigating the application effect of CP-modified CPI under stringent AF conditions possesses clear innovation and necessity.

Among the baked products, muffins are a popular choice for breakfast or afternoon snack, traditionally made from ingredients such as wheat flour, oil, eggs, sugar, and milk [22]. Developing AF muffins with superior sensory quality can not only meet the safety needs of allergic consumers but also significantly enhance their dietary experience and quality of life, holding significant practical application value. Therefore, this study used rice-based muffins as an AF prototype and investigated the preparation of AF muffins using CPI from three different sources (commercial CPI, laboratory CPI, and CP-treated laboratory CPI) at varying amounts. The aim is to examine the effects of adding CPI from different sources and in different amounts on the viscoelasticity and quality characteristics of the muffin batter and final products. This study not only highlights the innovative application of CP for modifying plant proteins in AF baked goods but also, through a systematic evaluation of the product’s physicochemical and textural properties, provides a feasible strategy and theoretical basis for developing nutritionally balanced, high-quality AF muffins that are acceptable to consumers.

## 2. Materials and Methods

### 2.1. Materials

Commercial chickpea protein (protein content: 83.57%) was purchased from Xi’an Endiyuan Biotechnology Co., Ltd. (Xi’an, China); sucrose and sodium bicarbonate were purchased from Zhanyi Baked Food Co., Ltd. (Shanghai, China); citric acid and carboxymethyl cellulose sodium (CMC) was purchased from Jiahe Xuri Co., Ltd. (Shenzhen, China); rice flour, canola oil, millet and salt were purchased from the local market (Ningbo, China).

Laboratory-made CPI was extracted using the alkaline extraction–isoelectric precipitation method as previously described [19]. Briefly, defatted chickpea flour was mixed with deionized water at a ratio of 1:10 (*w*/*v*), and the pH was adjusted to 9.0 using 1 M NaOH. The mixture was stirred at room temperature for 2 h, with pH readjustment every 30 min. After centrifugation at 7000 r/min for 15 min at 4 °C, the supernatant was collected and the extraction repeated once. The combined supernatants were adjusted to pH 4.5 using 1 M HCl to precipitate the proteins, followed by centrifugation. The precipitate was resuspended in deionized water, neutralized to pH 7.0, and freeze-dried. The protein content of the laboratory-made CPI was 85.26%. Laboratory-prepared CPI was treated for 30 s using a PSPT-JSPI-15 plasma generator (Nanjing Pustech Electronics Technology Co., Ltd., Nanjing, China). This treatment duration was determined as optimal based on our previous systematic evaluation of functional properties such as solubility, emulsifying capacity, foaming ability, and gelling capacity [19]. Air was used as the carrier gas, with flow rate and power set at 30 L/min and 680–700 W, respectively. The nozzle-to-sample distance was set to 40 mm. The protein content of CPI after CP treatment was 81.97%. Protein content was adjusted to the same level in all formulations. The differences between commercial and laboratory-made CPI may arise from variations in extraction processes, raw materials, and the degree of protein denaturation. Commercial CPI often undergoes intensive thermal processing and may contain additional components, leading to altered functional properties.

### 2.2. AF Muffin Batter Preparation

The preparation of AF muffin batter was based on the method proposed by Matos et al. [23] with minor modifications. with slight modifications. By incorporating 5%, 10%, and 15% of CPI, based on the weight of the rice flour, as a partial substitute for rice flour in the formulation, the effects of different CPI substitution ratios on AF muffin batter characteristics were explored. The specific formula is shown in Table 1. The preparation process for the AF muffin batters was as follows: Initially, water, sugar, citric acid, salt, and CMC were mixed at low speed for 3 min in a mixer (CE6001B Chang di mixer, Foshan WeiShiDa Electrical Industrial Co., Ltd., Foshan, China). Subsequently, rice flour, protein powder and sodium bicarbonate were added and blended at medium speed for 3 min. Finally, canola oil was incorporated and the mixture was beaten at high speed for 1 min. The batter was used for both the rheological test and to prepare the AF muffin.

The muffins without added protein are the NP group, and the muffins made with the addition of commercial CPI, laboratory-made CPI and cold plasma-processed laboratory-made CPI were named the CCPI group, LCPI group and CPLCPI group, respectively. The difference in the amount of protein added was because the concentrations of commercial CPI, laboratory-extracted CPI, and CP-processed laboratory-extracted CPI were 83.57%, 85.26%, and 81.97%, respectively.

### 2.3. Rheological Properties of AF Muffin Batters

Characterization of the rheological properties of AF muffin batters was performed on a DHR-2 rheometer (TA Instrument Co., New Castle, DE, USA) equipped with a parallel plate geometry of 60 mm diameter. The batter was placed between the two plates, the distance adjusted to 1 mm and excess sample scraped off, then the mixture was left for 5 min to obtain tension relaxation and temperature setting at 25 °C. The steady shear viscosity was acquired under a controlled shear rate from 1 to 100 s^−1^. Experimental data were described by a power law model as follows [22]:

*η* = *K·γ*^n−1^

where is apparent viscosity (Pa·s), *K* is the consistency coefficient (Pa·sn), *γ* is the shear rate (s^−1^), and n is the flow behavior index.

Stress sweep was run at 25 °C at constant frequencies of 1 Hz and strain was allowed to vary from 0.01% to 100% in order to determine the linear viscoelastic region (LVR). Frequency sweep tests were performed in the linear viscoelastic range (0.1% strain) from 0.01 to 10 Hz at 25 °C. To study the effect of heating in the batter structure, temperature sweeps were performed from 25 °C to 90 °C at a heating rate of 1.0 °C/min and a constant strain. Paraffin oil was applied to the exposed surfaces of all the samples, in order to prevent their drying during the measurements. The storage modulus (G′) and loss modulus (G″) were measured.

### 2.4. AF Muffin Preparation

The prepared muffin batters (Section 2.2) were then portioned into muffin cups, each weighing approximately 60 g. All batter samples were placed in an oven (Midea, Guangzhou, China) preheated to 180 °C and baked for 30 min at the same temperature. After baking, the muffins were allowed to cool to room temperature, then packaged in polypropylene bags and stored for one day until further analysis.

### 2.5. AF Muffin Properties

AF muffin height was measured with a digital vernier caliper for the highest point of muffin, and volume was measured with the millet seed replacement method. The baking loss of muffins after baking were calculated by subtracting the mass after baking from the mass before baking.

The internal and surface colors of the muffins were measured using a colorimeter (CR-400, Konica Minolta, Inc., Tokyo, Japan). The samples were positioned in front of the light source to record the L*, a*, and b* values. Here, the L* value indicates lightness, with a range from 0 to 100, where 0 is completely dark and 100 is completely light, reflecting the scale from dark to light. The a* value measures the spectrum between red and green; positive values denote a shift towards red, while negative values indicate a shift towards green. The b* value gauges the spectrum between yellow and blue, with positive values showing a tendency towards yellow and negative values suggesting a tendency towards blue. The L*a*b* values were obtained using Image J 2 software, thereby acquiring data for L* (lightness, ranging from 0 [black] to 100 [white]), a* (ranging from +60 [red] to −60 [green]), and b* (ranging from +60 [yellow] to −60 [blue]) [24].

Textural properties of muffin crumbs were measured using a texture analyzer (TA-XT +, Stable Micro Systems, Surrey, UK). The muffins were cut into squares (10 × 10 × 10 mm) from the center and then subjected to double compression (texture profile analysis) using a cylindrical probe equipped with a diameter of 100 mm. Compress 55% of the initial height at a speed of 2.0 mm/s [25]. Texture properties such as hardness, springiness, cohesiveness, chewiness, and resilience were calculated from the compression curve.

### 2.6. Micro-Structure of AF Muffin

Muffin crumbs were collected and freeze-dried using a vacuum freeze dryer (SCIENTZ-70FY Freeze-Dryer, Ningbo Scientz Biotechnology Co., Ltd., Ningbo, China). Subsequently, the microstructure of the muffin crumbs was observed using a scanning electron microscope (Hitachi S-3400, Tokyo, Japan).

### 2.7. Statistical Analysis

All experiments, including the rheological and texture profile analysis which fully followed the AACC standard procedures, were technical experiments conducted at least in triplicate (*n* ≥ 3). Statistical analysis was carried out using SPSS 26.0 statistical software, and the collected data met all assumptions of analysis of variance (ANOVA), including normality, homogeneity of variance, and independence of observations. The significance of samples was statistically analyzed by one-way analysis of variance (ANOVA) with Duncan’s multiple range test, and the significance level was set to *p* < 0.05.

## 3. Results

### 3.1. Rheological Properties of Batters

#### 3.1.1. Apparent Viscosity

**Figure 1:**
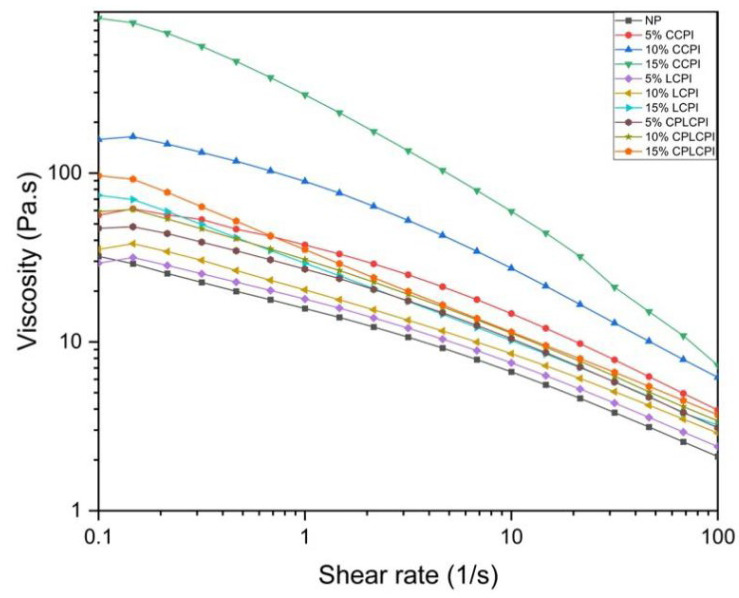
The apparent viscosity of AF muffin batter made by adding CPI from different sources and different contents.

Apparent viscosity provides insight into how the ingredients affect the rheological behavior of muffin batter. The flow curves of AF muffin batters with added protein are presented in Figure 1, where the shear rate was controlled within the range of 1–100 s^−1^. All samples demonstrated increased levels of apparent viscosity upon the addition of protein. This increase was likely attributable to the protein’s strong water-binding capacity, which helps to retain and more effectively lock in water during flow [26,27,28]. Table 2 displayed the power law parameters for all muffin batter samples, showing that the data fit the power law model effectively. The flow index (n) varied between 0.48 and 0.69, confirming that all muffin batters displayed shear-thinning (pseudoplastic) behavior, especially at shear rates of 10–50 s^−1^. The elevated flow index may be attributed to the presence of CMC in the sample formulation, aligning with findings from the study by Demirkesen et al. [29]. Compared to the NP group without protein addition, the maximum consistency index (K) increased from 16.28 ± 1.69 Pa·sn to 295.27 ± 15.40 Pa·sn, indicating that CPI addition significantly enhanced the apparent viscosity of the batter. Commercial CPI contains multiple types of proteins and exhibits high apparent viscosity during flow. This is likely due to the higher degree of thermal denaturation experienced during commercial production, which causes protein unfolding and intermolecular aggregation, thereby forming a stronger network structure. The CP-treated laboratory CPI exhibited a higher *K* and a lower n compared to the NP group without added protein. This suggests that the addition of CP-treated laboratory CPI strengthens the shear-thinning behavior of the batter. This effect could be attributed to the protein’s interaction with other components in the batter, which enhances its flexibility and reduces its tendency to deform during flow. Furthermore, CP treatment may expose polar groups (such as -OH and -NH2), which could form a hydrogen bond network with rice starch. The study found that changes in the structure of this hydrogen bond network further affect the viscosity of the batter [30].

When CPI was treated with CP, the spatial structure of the protein was altered, and the amino acid side chains expanded [19]. During the mixing and stirring process with ingredients such as rice flour, water, and sugar, the protein was better able to integrate and form a stable structure, thereby effectively retaining moisture. However, adding an excessive amount of protein can increase the viscosity of the muffin batter, likely because the protein binds a large amount of water, resulting in a stiffer batter base. Adjusting the viscosity of the batter is crucial because it affects the stability and expansion of the air chamber during awakening, which affects the muffin quality. Meanwhile, the viscosity and thickness of the batter play a crucial role in determining the volume of the muffin. However, higher viscosity and thickness can limit the expansion of the batter during baking, resulting in a smaller volume [31].

#### 3.1.2. Frequency Sweep

**Figure 2:**
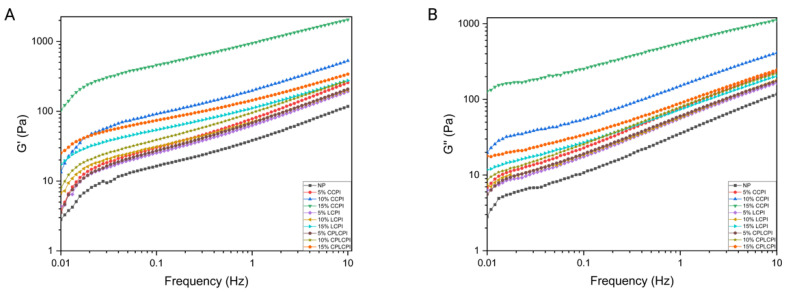
The frequency sweep of AF muffin batter made by adding CPI from different sources and different contents. (**A**) (G’), (**B**) (G″).

Figure 2A,B present the G’ and G″ of AF muffin batters made by adding different types and amounts of CPI. The graphs revealed that the curve shapes for all samples were quite similar, exhibiting typical soft gel behavior with G’ higher than their viscous counterparts. This indicated that all AF muffin batters have tangent loss values below 1, reflecting a predominantly elastic behavior. Furthermore, for all samples, both the G’ and G″ increased with rising frequency, demonstrating frequency-dependent behavior. Batters with a higher protein content displayed increased G’ and G″, likely due to the protein’s stiffening effect on the batter structure [32]. Moreover, previous studies, including those by Marcoa & Rosell [33], have indicated that CPI possesses a high water-binding capacity, which contributes to the increased viscosity observed in the batters. When the ingredients used in the muffin batter have a high water-holding capacity, it reduces free water and particle movement, thus promoting an increase in the muffin batter viscosity [34]. This finding aligns with observations that the inclusion of soy protein isolate significantly altered the viscoelastic properties of rice dough, further highlighting the impact of protein type and concentration on the rheological behavior of food matrices.

AF pancake batter prepared with commercial CPI exhibited the highest viscoelasticity, consistent with its high *K* value in apparent viscosity testing. This collectively confirms that its highly aggregated or denatured proteins formed a stronger network structure.

In contrast, the relatively lower initial moduli observed in laboratory-prepared CPI reflect the preservation of its “near-native” state through the gentle extraction process. The protein molecules of this CPI are well-dispersed and free from pre-aggregation, thus eliminating the need to rely on pre-existing physical cross-linking points for network formation. Instead, the hydrated protein molecules must gradually construct a dynamic yet initially weak foundational network by forming slow intermolecular interactions with surrounding components. This intact and undenatured characteristic effectively maintains the integrity of its active sites, thereby providing a superior substrate for subsequent cold plasma (CP) treatment.

In addition, the viscoelastic changes in muffin batters made with CP-treated CPI were also studied. Compared with the muffin batters made from CPI without CP treatment, the batters made from CPI after CP treatment showed higher viscoelasticity. This may be due to the fact that CP treatment accelerated protein unfolding and increases the protein’s ability to bind water, thereby producing a stronger batter matrix [19,35]. This aligns with our previous observation that CP treatment enhances protein hydration and network formation capabilities, indicating that the addition of protein significantly increases the viscoelasticity of the batter.

#### 3.1.3. Temperature Sweep

**Figure 3:**
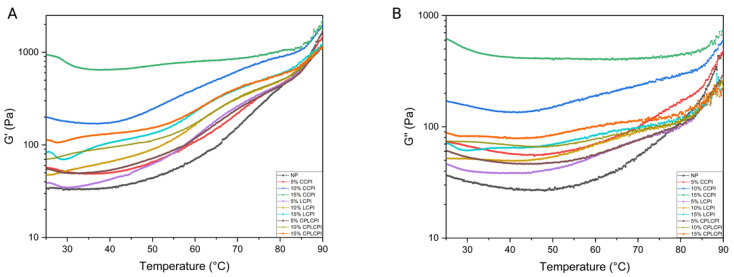
The temperature sweep of AF muffin batter made by adding CPI from different sources and different contents. (**A**) (G’), (**B**) (G″).

To investigate the impact of protein type and the amount added on AF muffin batters during the heating process, a rheometer was employed to monitor viscoelastic changes via temperature scanning. The alterations in the G’ and G″ of the AF muffin batters as they were heated from 25 °C to 90 °C are depicted in Figure 3A,B. AF muffin batters with added protein displayed higher values of G’ and G″, indicating that the addition of protein improved the elasticity and mechanical strength of the batter [35].

During the initial phase of heating, AF muffin batters with added protein exhibited a significant decrease in G’ and G″. This reduction was likely due to the unfolding of proteins early in the heating process, which led to a gradual loss of protein strength and a decrease in the G’ [36,37]. As the temperature continued to rise, interactions occur among ingredients such as rice flour, proteins, and CMC, forming a heat-induced gel. This created a firm network to maintain the structure of the batter, resulting in increases in both G’ and G″. This behavior may also be related to the specific structure of the proteins involved. Compared to modified soy protein and pea protein, CP-modified CPI exhibits unique advantages in enhancing the viscoelasticity of rice paste. This may be attributed to CPI’s distinctive 7S and 11S globulin composition and its specific conformational changes under CP modification. During the heating process, oligomers of these proteins dissociate, exposing more chemical groups, which increases the opportunities for binding with rice flour and CMC, forming a more stable gel network [38,39]. As the temperature approached 85 °C, there was a notable sharp rise in the G’ slope for batters with added protein, indicative of CPI gelation occurring at this temperature. This transformation contributed to the enhanced viscoelastic properties of the batter, supporting the formation of a stable, gel network structure.

The AF pancake batter containing CP-treated CPI exhibited higher G’ and G″ values throughout the heating process. This increase can be linked to the unfolding of protein structures caused by CP treatment. The exposed hydrophobic groups from this unfolding enhanced the interactions with rice flour and other components in the batter, contributing to the improved viscoelastic properties observed [19].

### 3.2. Height, Volume and Baking Losses of AF Muffins

There were significant differences in the volume, weight loss and height of AF muffins made with different types and quantities of CPI, as shown in Table 3 (*p* < 0.05). As the amount of protein added increases, so does the volume of the muffin. It is worth noting that the muffin made with 15% CPI treated with CP had the largest volume of 99.43 cm^3^, which was 20.1% higher than that of the NP group (82.77 cm^3^). This can be attributed to the fact that the CP process may enhance the flexibility of proteins by unfolding amino acid side chains, thereby improving foam formation at the water–air interface and maintaining foam stability. This enables the batter to retain more carbon dioxide when mixed with other ingredients, leading to the formation of air pockets and thus increasing the batter volume [40]. In addition, during the roasting process, as the temperature increases, the protein is denatured and its structure unfolds, resulting in the formation of more disulfide bonds. This interaction with rice flour, sugar, and oil contributes to the formation of a more stable gel network that supports the volume of muffins [41]. Conversely, the size of the muffins in the 15% LCPI group was significantly smaller than that in the 10% LCPI group. This reduction may be attributed to the high protein content, which causes excessive protein aggregation during heating. This aggregation disrupts the gel network and reduces the batter’s ability to retain carbon dioxide, ultimately leading to a decrease in volume.

**Figure 4:**
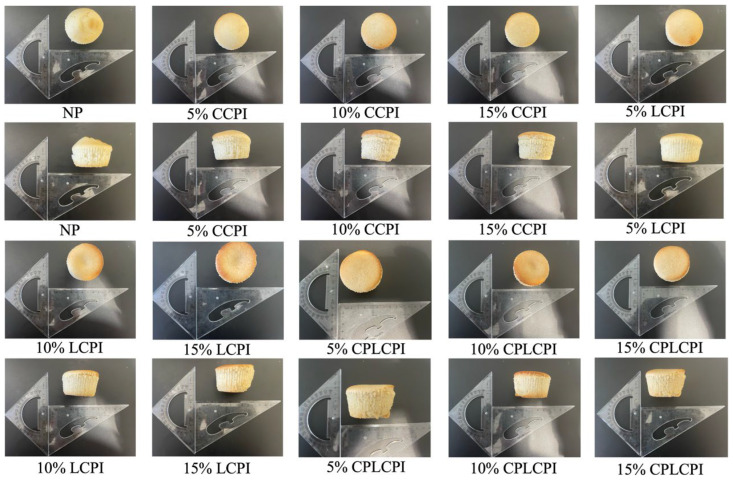
Digital photos of AF muffins.

The weight loss of muffins is often referred to as baking loss, and the main reason is the evaporation of water during the baking process. We observed a downward trend in muffin weight loss with the addition of protein, especially in the 10% LCPI, 5% CPLCPI, and 10% CPLCPI groups. The reduction in weight loss can be attributed to the higher water-holding capacity of the CP, which retains moisture more efficiently and thus reduces the evaporation rate during the roasting process [42]. In addition, during the baking process, the CMC and CPI in the batter help to form a gel network in the batter, which locks in moisture and reduces baking losses. Conversely, we found that the height of the muffins decreased significantly with the addition of protein (*p* < 0.05). This may be because muffins with no added protein tend to rise more in the center, resulting in higher peaks, while muffins with added protein are more evenly distributed in height. Essentially, protein-rich muffins don’t exhibit the same central peak, resulting in an overall shorter stature. This effect on volume and height can also be seen in the visual observation of the muffins (Figure 4).

### 3.3. Color Parameters of AF Muffins

**Figure 5:**
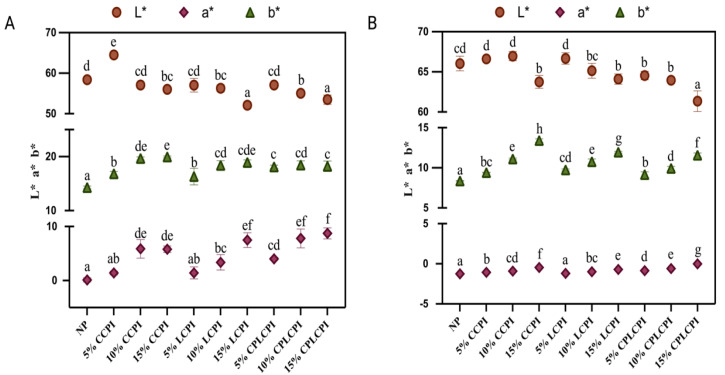
Color parameters of AF muffins made by adding CPI from different sources and different contents. (**A**) (Surface), (**B**) (Internal). Different letters for the same indicator indicate significant differences (*p* < 0.05).

The color of a muffin is a key characteristic that consumers immediately notice, significantly influencing their preferences and choices. The color parameters of AF muffins were markedly influenced by the type and amount of protein powder used. The changes in the surface and internal colors of AF muffins are depicted in Figure 5A,B. Following the incorporation of CPI, the L* value of the AF muffins notably decreased, a finding aligned with Matos et al. [23]. Furthermore, an increase in protein content resulted in a further decrease in the L* value, probably due to the inherent darker color of CPI. The L* value was closely tied to the original color of the protein and inversely related to the amount of protein added. During baking, the product’s color may result from the raw materials’ inherent colors or their interactions, such as the Maillard and caramelization reactions [43,44]. AF muffins made with CP-treated CPI exhibited lower L* values, possibly because the CP treatment unfolded the protein structure, exposing more amino acid side chains, enhancing interactions with reducing sugars, and thus promoting the Maillard reaction, which affected the muffin’s color change [45]. Concurrently, the a* and b* values of the AF muffins increased significantly with added protein, attributable to the pigments in CPI altering the muffin’s color.

### 3.4. Textural Properties of AF Muffins

**Figure 6:**
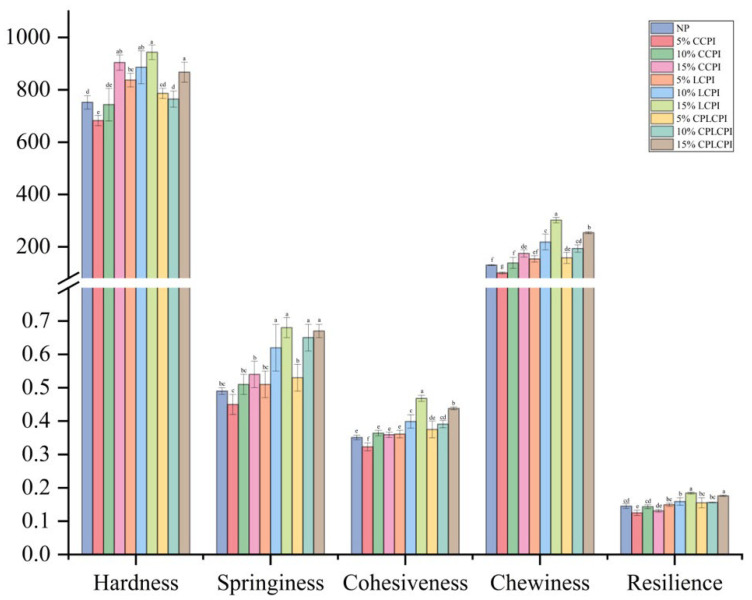
Texture parameters of AF muffins made by adding CPI from different sources and different contents. Different letters for the same indicator indicate significant differences (*p* < 0.05).

The type and amount of protein significantly affect the texture parameters of AF muffins (*p* < 0.05), as detailed in Figure 6. Parameters such as hardness, springiness, cohesiveness, chewiness, and resilience were assessed through force-versus-time measurements, with changes in these parameters directly influencing the muffin’s mouthfeel and texture.

The hardness of the control muffin was measured at 752.22 g. Adding protein significantly increased the hardness of the AF muffins, particularly at higher protein concentrations. This effect may be attributed to the interactions between protein and water during baking, which retain substantial amounts of free water, thereby reducing the muffin’s flexibility [45]. Increases in springiness and cohesiveness were associated with freshness, aeration, and a bouncy texture in muffins, while greater cohesiveness indicated less friability [46]. According to Figure 6, the springiness and cohesiveness of AF muffins significantly increase with increasing protein content. This correlates the higher viscosity seen in muffin batters with more protein, which during baking led to protein gelation and the formation of an elastic network that holds air [47,48]. Chewiness, a texture parameter that influences chewing ease, follows a similar trend to hardness, signifying a denser cake structure and enhanced resistance to chewing. Supporting previous findings, the inclusion of chickpea, pea, lentil, and broad bean flours significantly raised the hardness and chewiness of rice cakes [44]. Additionally, increases in springiness, cohesiveness, and resilience reflected a more aerated structure within the muffins, correlating positively with their overall quality.

In the rheological analysis of muffin batter, it was observed that batter containing commercial CPI exhibited higher viscosity, elasticity, and thickness. However, the muffins baked with this batter displayed higher hardness and lower elasticity, which may be due to the high degree of denaturation of the commercial CPI. The extensive disruption of its original structure leads to the formation of a gel network that restricts the expansion of the muffin batter. In addition, compared with the AF muffins in the 15% CCPI group and the 15% LCPI group, the AF muffins in the 15% CPLCPI group had lower hardness, higher springiness, and higher cohesiveness. This behavior was likely due to the CP treatment facilitating the unfolding of the protein’s spatial structure, exposing more binding sites. During baking, these proteins more readily interacted with other ingredients, forming a stable, elastic network that captures more air. Consequently, using CP-treated CPI not only addressed protein deficiency but also markedly enhanced the textural properties of the muffin. This result outperforms the performance of pea protein or lentil protein in similar applications reported in some studies. For instance, research indicates that pea protein requires blending with starch to effectively improve texture [49], while lentil protein alone may result in excessively hard products [47]. In this study, CPLCPI achieved a favorable balance of hardness and elasticity without requiring additional starch support, highlighting its potential as a high-quality structural protein in AF baking systems.

### 3.5. Micro-Structure of AF Muffins

**Figure 7:**
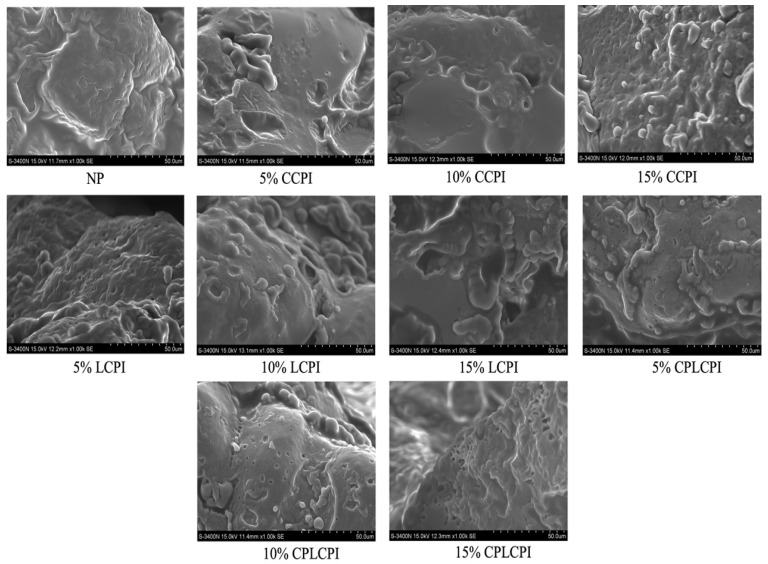
Micro structure of AF muffins made by adding CPI from different sources and different contents.

Scanning electron microscopy was used to evaluate the microstructural changes in AF muffin grains supplemented with CPI, as shown in Figure 7. The images show that the AF muffins without CPI have a denser and uneven pore structure, which is consistent with the typical characteristics of traditional protein-free baked products [50]. In contrast, the surface of the AF muffin sample with the addition of protein showed a large number of pores. This can be attributed to protein cross-linking, forming a structural backbone capable of preserving the bubble structure [51]. Further analysis showed that the pore density on the surface of AF muffins was closely related to the type and amount of protein added. The higher protein content allows for more interaction with other ingredients during the baking process, resulting in a stable framework that effectively retains air bubbles and prevents the muffin structure from collapsing [52].

Notably, the surface of AF muffins made with commercial CPI showed smaller pores and thicker pore walls. This may be due to the complete denaturation of the protein, which inhibits the formation of a stable skeleton that can retain pores during the baking process, thus limiting batter expansion. In contrast, muffins made with CPI-treated CPI have a uniformly distributed porous structure with thin and continuous pore walls. CP treatment enhances the interaction with rice flour and CMC by unfolding the hydrophobic group of the protein to form a stable three-dimensional network [42]. This structural modification helps to maintain the pore structure during the mixing and baking phases, while giving the AF muffins a higher elasticity.

## 4. Conclusions

In this study, CPI was added as a protein supplement to AF rice-based muffins. The results demonstrated that the addition of CPI improved the viscoelasticity and thickness of the AF muffin batter. The muffins produced were larger in volume, had better color and texture, and exhibited more porous structures in their microstructure. Furthermore, the quality of muffins prepared with different types and concentrations of CPI showed significant differences (*p* < 0.05). Due to the severe denaturation of commercial CPI, the muffin batter prepared with it exhibited higher viscosity and thickness, which limited the expansion of the muffins during baking. The muffins produced had relatively poor volume, height, and textural properties. Treating CPI with CP caused the protein structure to unfold, thereby enhancing its functional properties. During baking, this treatment increased the protein binding capacity with other ingredients and formed a stable framework to retain air, resulting in higher-quality muffins. Additionally, muffins made with 15% CP-treated CPI were larger, more elastic, and exhibited improved color. This study integrates CP technology, CPI, and AF products, providing a theoretical foundation for developing AF muffins with balanced nutrition, superior texture, and enhanced quality. Nevertheless, it should be noted that the absence of systematic sensory evaluation constitutes a significant limitation. The ultimate acceptance of a product depends not only on instrumentally measured texture and color, but more critically on consumers’ sensory experience. Future research should focus on the application of modified CPI in different AF flour systems and the analysis of its flavor characteristics, while further evaluating its sensory acceptance and shelf life to advance the industrial application of this technology.

## Figures and Tables

**Table 1:** Ingredients used in allergen-free muffin recipe (g).

	Rice Flour	Water	Sugar	CPI	Canola Oil	Sodium Bicarbonate	Citric Acid	Salt	CMC
NP (No Protein)	100	110	75	0	46	4	3	1.5	1
5%CCPI (Commercial CPI, 5% addition)	94	110	75	6	46	4	3	1.5	1
10%CCPI (Commercial CPI, 10% addition)	88	110	75	12	46	4	3	1.5	1
15%CCPI (Commercial CPI, 15% addition)	82	110	75	18	46	4	3	1.5	1
5%LCPI (Laboratory CPI, 5% addition)	94.1	110	75	5.9	46	4	3	1.5	1
10%LCPI (Laboratory CPI, 10% addition)	88.2	110	75	11.8	46	4	3	1.5	1
15%LCPI (Laboratory CPI, 15% addition)	82.3	110	75	17.7	46	4	3	1.5	1
5%CPLCPI (CP-Treated Laboratory CPI, 5% addition)	93.9	110	75	6.1	46	4	3	1.5	1
10%CPLCPI (CP-Treated Laboratory CPI, 10% addition)	87.8	110	75	12.2	46	4	3	1.5	1
15%CPLCPI (CP-Treated Laboratory CPI, 15% addition)	81.7	110	75	18.3	46	4	3	1.5	1

**Table 2:** Power law model parameters of AF muffin batters made with different sources and different levels of CPI.

	*K* (Pa·sn)	n	r2
NP	16.28 ± 1.69 f	0.67 ± 0 bc	0.995
5% CCPI	34.85 ± 1.53 c	0.69 ± 0.006 ab	0.962
10% CCPI	82.48 ± 3.33 b	0.69 ± 0.006 a	0.982
15% CCPI	295.27 ± 15.4 a	0.64 ± 0 de	0.984
5% LCPI	18.11 ± 1.95 ef	0.67 ± 0.007 bc	0.977
10% LCPI	19.92 ± 0.62 def	0.63 ± 0.011 e	0.981
15% LCPI	31.43 ± 3.06 cd	0.54 ± 0.004 h	0.995
5% CPLCPI	24.88 ± 0.26 cdef	0.57 ± 0.004 f	0.977
10% CPLCPI	28.93 ± 0.3 cde	0.48 ± 0.009 i	0.982
15% CPLCPI	35.13 ± 0.88 c	0.65 ± 0.006 cd	0.993

Different letters for the same indicator indicate significant differences (*p* < 0.05).

**Table 3:** Physical characteristics of AF muffins made by adding CPI from different sources and dif ferent contents.

	Volume/cm^3^	Weightloss/g	Height/mm
NP	82.77 ± 0.42 f	6.78 ± 0.44 a	42.02 ± 0.68 a
5% CCPI	87.33 ± 0.60 d	6.50 ± 0.35 abcd	40.24 ± 0.49 c
10% CCPI	92.73 ± 0.35 b	6.70 ± 0.26 ab	40.86 ± 0.13 bc
15% CCPI	93.70 ± 0.44 b	6.23 ± 0.38 abcd	39.23 ± 0.20 d
5% LCPI	84.93 ± 0.76 e	6.78 ± 0.38 a	36.79 ± 0.31 fg
10% LCPI	88.97 ± 0.70 c	6.08 ± 0.22 cd	37.18 ± 1.00 f
15% LCPI	82.13 ± 0.38 f	6.60 ± 0.36 abc	38.10 ± 0.85 e
5% CPLCPI	83.10 ± 0.36 f	6.15 ± 0.41 bcd	36.02 ± 0.29 g
10% CPLCPI	79.27 ± 0.96 g	5.98 ± 0.41 d	36.83 ± 0.43 fg
15% CPLCPI	99.43 ± 0.61 a	6.20 ± 0.42 abcd	41.23 ± 0.74 ab

Different letters for the same indicator indicate significant differences (*p* < 0.05).

## Data Availability

The data are avaliable from the corresponding author.

## Abbreviations

The following abbreviations are used in this manuscript:

AF	Allergen-free
CPI	Chickpea protein isolate
FA	Food allergies
CP	Cold plasma
CMC	Carboxymethyl cellulose sodium
LVR	Linear viscoelastic region
